# The emerging role of septins in fungal pathogenesis

**DOI:** 10.1002/cm.21765

**Published:** 2023-06-02

**Authors:** Iris Eisermann, Marisela Garduño‐Rosales, Nicholas J. Talbot

**Affiliations:** ^1^ The Sainsbury Laboratory University of East Anglia Norwich UK

**Keywords:** appressorium, cell polarity, fungi, invasive growth, plant pathogens, plant penetration, septins

## Abstract

Fungal pathogens undergo specific morphogenetic transitions in order to breach the outer surfaces of plants and invade the underlying host tissue. The ability to change cell shape and switch between non‐polarised and polarised growth habits is therefore critical to the lifestyle of plant pathogens. Infection‐related development involves remodelling of the cytoskeleton, plasma membrane and cell wall at specific points during fungal pathogenesis. Septin GTPases are components of the cytoskeleton that play pivotal roles in actin remodelling, micron‐scale plasma membrane curvature sensing and cell polarity. Septin assemblages, such as rings, collars and gauzes, are known to have important roles in cell shape changes and are implicated in formation of specialised infection structures to enter plant cells. Here, we review and compare the reported functions of septins of plant pathogenic fungi, with a special focus on invasive growth. Finally, we discuss septins as potential targets for broad‐spectrum antifungal plant protection strategies.

## 1 INTRODUCTION

Fungal pathogens cause many of the most devastating diseases in plants, leading to significant economic losses and endangering the nutrition of at least 3 billion people worldwide (Brown & Wilby, 2012; FAO, 2018; Fisher et al., 2016). Fungal pathogens have developed different strategies to infect and colonise plant tissues, and to propagate in the field. One key strategy of fungal pathogens is their capacity to undergo morphological changes involving a switch from polarised to non‐polarised growth and vice versa. This results in the development of structures such as appressoria and hyphopodia to breach the tough outer surfaces of plants and haustoria to invade plant cells directly. These cell shape changes require remodelling of the cytoskeleton, and morphogenetic proteins called septins play a major role in regulating these cell shape changes during infection‐related development.

Septins are small GTPases that were first discovered to play a role in cytokinesis in the budding yeast *Saccharomyces cerevisiae* (Hartwell, 1971). In many eukaryotic organisms, septins are involved in cellular organisation processes, such as polarity determination, secretion, cytokinesis, neuronal function and endocytosis. Their diverse functions may stem at least in part from their ability to sense micron‐scale plasma membrane curvature and assemble at membranes both at the cortex and within cells (Bridges et al., 2016; Dagdas et al., 2012; Douglas et al., 2005; Momany et al., 2001; Spiliotis & Nelson, 2006). Septins form hetero‐oligomeric complexes that interact with a wide variety of other proteins—including membrane‐associated proteins implicated in polarity generation and alteration of membrane properties (Bridges et al., 2016; Dagdas et al., 2012; Momany et al., 2001; Spiliotis & Nelson, 2006). Septins that have been shown to form hetero‐oligomeric complexes are often termed core septins and are orthologues of Cdc3, Cdc10, Cdc11 and Cdc12 originally identified in *S. cerevisiae* (Hartwell, 1971). In addition to core septins, some filamentous fungi and protists also possess “non‐core” septins (Pan et al., 2007). Septins have recently been classified into five different classes and are highly conserved across eukaryotes, except in multicellular plants where they are conspicuously absent (Shuman & Momany, 2021). Septins contain an N‐terminal polybasic domain that binds to phosphoinositides at the plasma membrane (Casamayor & Snyder, 2003; Leipe et al., 2002), which is followed by a GTP‐binding domain and a septin unique element, that distinguishes septins from other P‐loop‐containing GTPases (Pan et al., 2007; Versele & Thorner, 2004). A coiled‐coil domain is located at the C‐terminus of septins (Casamayor & Snyder, 2003; Pan et al., 2007). These domains allow septins to form higher‐order structures, such as bars/rods, gauzes, discs and rings (Bridges & Gladfelter, 2015). Higher‐order structures confer the capability to facilitate dramatic remodelling events, such as cellular constrictions, reorientation of the axis of growth, branching of filamentous cells and cortical rigidity (DeMay et al., 2011; Dulal et al., 2020).

## 2 SEPTINS IN FILAMENTOUS ASCOMYCETE FUNGI AND THEIR ROLE IN PLANT PATHOGENESIS

### 2.1 How do septins contribute to the development of infection structures in fungal plant pathogens?

The role of septins in fungal pathogenesis has been studied in taxonomically diverse plant pathogens. The rice blast fungus *Magnaporthe oryzae* is a filamentous ascomycete and causal agent of the most serious disease of cultivated rice, destroying enough rice each year to feed 60 million people (Skamnioti & Gurr, 2009). The blast fungus infects host plants using a dome‐shaped, melanin‐pigmented infection structure known as an appressorium, which enables protrusion of a narrow, rigid penetration peg to rupture the rice leaf cuticle (Wilson & Talbot, 2009). Plant infection involves turgor‐mediated isotropic expansion of the appressorium, coupled with tight adhesion of the infection structure to the leaf surface. This is followed by re‐polarisation resulting in emergence and growth of a rigid penetration peg that ruptures the cuticle and plant cell wall (Ryder et al., 2019). The penetration peg does not, however, rupture the plant plasma membrane and instead this becomes invaginated around the invasive hypha as it further develops by budding growth to fill the initial epidermal cell. Appressorium development in *M. oryzae* requires the Pmk1 MAP kinase pathway, which responds to surface signals to induce MAPK phosphorylation and activation of a hierarchical transcriptional network that regulates appressorium morphogenesis via a series of transcription factors, including the Hox7 homeobox protein, and appressorium maturation via the Mps1 regulator. Collectively, and likely through the action of many other transcriptional regulators, appressorium‐mediated plant infection is facilitated by this conserved signalling pathway (Osés‐Ruiz et al., 2021; Sakulkoo et al., 2018). Appressorium morphogenesis is also accompanied by autophagic cell death of the conidium from which the appressorium develops. This process is necessary to enable trafficking of the contents of the three‐celled spore into the appressorium to generate the enormous turgor required to produce the protrusive force to breach the cuticle.

**FIGURE 1:**
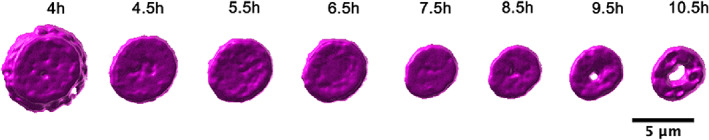
Temporal dynamics of septin ring assembly during appressorium development in *M. oryzae*. A conidial suspension of Sep5‐RFP strain of *M. oryzae* at 5 x 10^4^ mL^−1^ was inoculated onto glass coverslips and images were taken at eight different time points post‐inoculation. The 3D rendered images generated in Imaris (9.5.1; Bitplane), show the Sep5‐containing septin disc at the base of the appressorium reaching its biggest volume at 4 h. Afterwards, the septin disc contracts until it starts to form a septin ring at 9.5 h. Scale bar = 5 μm. Image courtesy of Dr. Alice Bisola Eseola.

**FIGURE 2:**
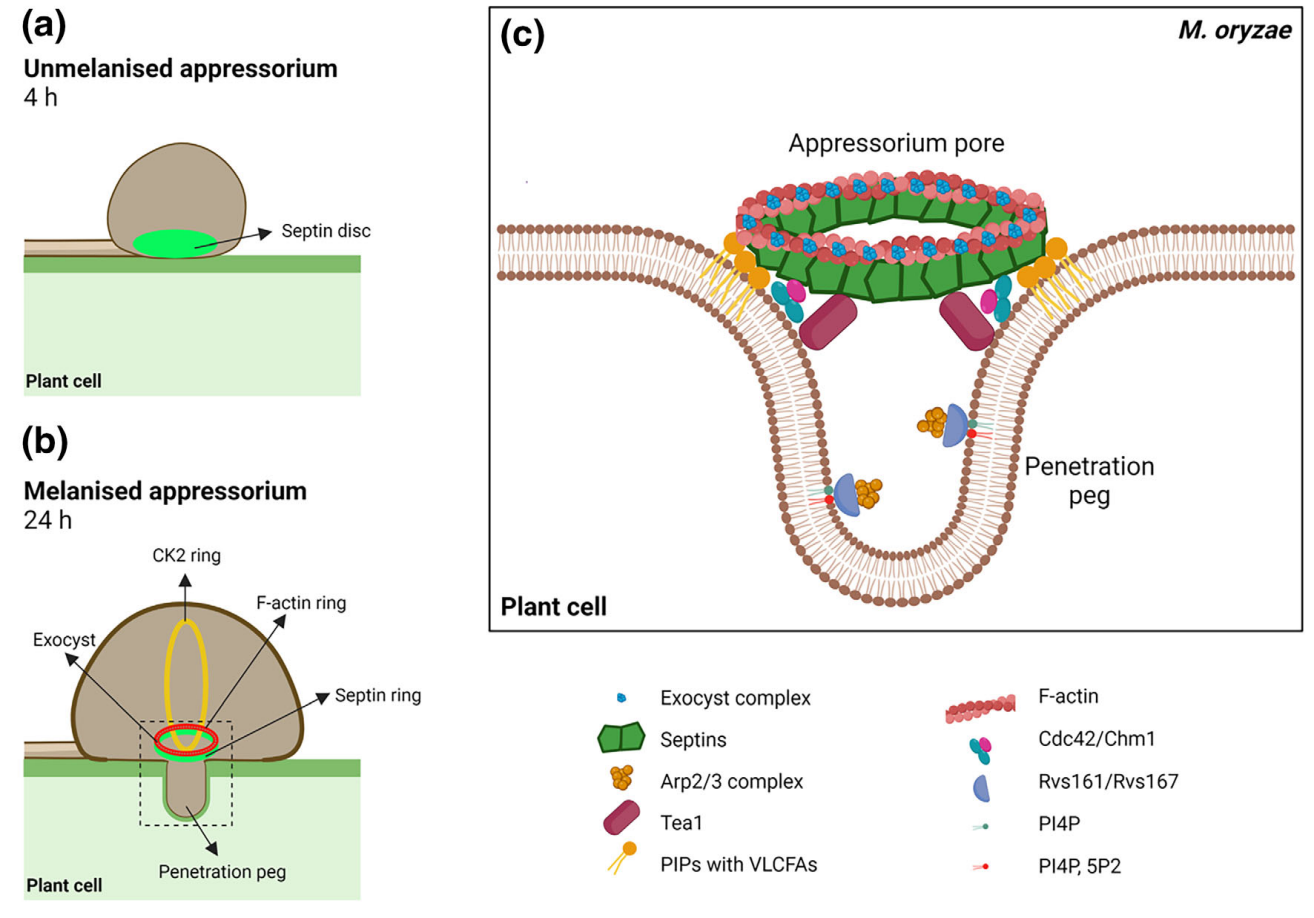
Model of the septin‐mediated rice cuticle penetration by *Magnaporthe oryzae*. (a) Initial non‐melanised appressorium at 4 h post inoculation, harbouring the septin disc. (b) A melanised appressorium at maximal turgor displaying the septin‐, actin‐ and CK2‐ring at 24 h post‐inoculation. (c) Close‐up view of the penetration peg at the base of the appressorium, showing the septin‐ring and known interactors. Adapted from Cruz‐Mireles et al. (2021).

Septins play an essential role in pathogenesis of *M. oryzae*. The fungus possesses six septins in total, and the four core septins—Sep3, Sep4, Sep5 and Sep6 (see Table 1)—form a hetero‐oligomeric disc‐like structure which then contracts into a ring of approximately 5 μm in diameter at the base of the appressorium (Dulal et al., 2020) (see Figures 1 and 2). This region, called the appressorium pore, is clearly differentiated from the rest of the infection structure and lacks melanin or a thickened cell wall. Septins scaffold F‐actin to form a toroidal network at the base of the appressorium, around the pore (Dagdas et al., 2012; Dulal et al., 2020). In septin deletion mutants the F‐actin network fails to organise at the appressorium pore and, as a consequence, no penetration peg forms and the mutants are non‐pathogenic. Septin deletion mutants also mis‐localise proteins involved in endocytosis, such as Rvs167, and actin re‐organisation such as gelsolin and Tea1 (Dagdas et al., 2012) (see Figure 2). Dulal et al. (2020) have proposed that during septin disc emergence, septin rods and filaments undergo annealing through diffusion and collision at the plasma membrane at the base of the nascent appressorium, thereby forming the ring conformation. They also demonstrated how the microtubule cytoskeleton is also organised in a septin‐dependent manner with microtubules arranged perpendicular to the actin and septin ring (Dulal et al., 2020; Gupta et al., 2015; Yan et al., 2022). Two non‐core septins, Sep7 and Sep8 are also present in *M. oryzae* (Table 1) but their precise role in development and pathogenesis awaits further characterisation.

**TABLE 1:** Characterised (bold) and uncharacterised septins of a sub‐set of plant pathogenic fungi compared to those in the budding yeast *Saccharomyces cerevisiae*.

Fungal species	Group^a^				
	Core septins				Non‐core septins
	2a	1a	3	4	5 and septin‐like
*Botrytis cinerea*	Cdc3^b^	**Sep4**	Cdc11	Cdc12	AspE, AspE2
*Bipolaris maydis*	**Cdc3**	**Cdc4**	**Cdc5**	**Cdc6**	**Cdc100**, Cdc101
*Colletotrichum gloeosporioides*	Cdc3	**Sep4**	Cdc11	Cdc12	AspE, AspE2
*Colletotrichum orbiculare*	Cdc3	Cdc10	Cdc11	**Sep6**	AspE, AspE2
*Cryphonectria parasitica*	Cdc3	Cdc10	**Sep1**	Cdc12	AspE, AspE2
*Fusarium graminearum*	**Cdc3**	**Cdc10**	**Cdc11**	**Cdc12**	AspE, AspE2, Hyp7A
*Magnaporthe oryzae*	**Sep3**	**Sep4**	**Sep5**	**Sep6**	**Sep7, Sep8**
*Ustilago maydis*	**Sep1**	**Sep4**	**Sep3**	**Sep2**	
*Verticillium dahliae*	**Sep3**	**Sep4**	**Sep5**	**Sep6**	AspE, AspE2
*Saccharomyces cerevisiae*	**Cdc3**	**Cdc10**	**Cdc11** **Shs1** **Spr28**	**Cdc12** **Spr3**	

Fungus	Group				
	Core septins				Non‐core septins
	2a	1a	3	4	5 and septin‐like
*Botrytis cinerea*	Sep3	**Sep4**	Sep5	Sep6	Sep7, Sep8
*Bipolaris maydis*	**Cdc3**	**Cdc4**	**Cdc5**	**Cdc6**	**Cdc100**, Cdc101
*Colletotrichum gloeosporioides*	Sep3	Sep4	Sep5	Sep6	Sep7, Sep8
*Colletotrichum orbiculare*	Sep3	Sep4	Sep5	**Sep6**	Sep7, Sep8
*Cryphonectria parasitica*	Sep3	Sep4	**Sep1**	Sep6	Sep7, Sep8
*Fusarium graminearum*	**Cdc3**	**Cdc10**	**Cdc11**	**Cdc12**	AspE, AspE2, Hyp7A
*Magnaporthe oryzae*	**Sep3**	**Sep4**	**Sep5**	**Sep6**	Sep7, Sep8
*Ustilago maydis*	**Sep1**	**Sep4**	**Sep3**	**Sep2**	
*Verticilium dahliae*	**Sep3**	**Sep4**	**Sep5**	**Sep6**	Sep7, Sep8
*Saccharomyces cerevisiae*	**Cdc3**	**Cdc10**	**Cdc11** **Shs1** **Spr28**	**Cdc12** **Spr3**	

^a^ Groups as defined by Shuman and Momany (2021).

^b^ Uncharacterised septins are yet to be named and referenced based on exemplars from each group defined by Shuman and Momany (2021).

The recruitment of septins to the appressorium pore also requires the action of a Ras GTPase‐activating protein, Smo1, which interacts with autophagy proteins Atg3, Atg4, Atg5 and Atg7, in addition to Sep3, Sep4, Sep5, Sep6 and Sep7 (Kershaw et al., 2019). Smo1 was originally identified as a mutant in which conidia were spherical and only one or two‐celled (Hamer et al., 1989). In addition, Smo1 mutants were affected in appressorium morphogenesis and pathogenicity. Smo1 was subsequently demonstrated to be a RAS GAP protein that likely plays a role in the orchestration of cytoskeletal remodelling. Autophagy plays a fundamental role in appressorium development in *M. oryzae* (Veneault‐Fourrey et al., 2006) and appears to be a Pmk1‐dependent process (Osés‐Ruiz et al., 2021) that must be temporally co‐ordinated with septin aggregation. In *Bipolaris maydis* (also known as *Cochliobolus heterostrophus*), the causal agent of southern‐corn leaf blight disease, the autophagy genes *ATG4* and *ATG8* are necessary for reproductive development, virulence and septin assembly (Yu et al., 2022). Impairment in autophagy causes disordered Cdc10 subcellular localisation and prevents septin ring formation in appressoria (Yu et al., 2022). This is particularly interesting because Cdc10 mutants are the only septin mutants in *B. maydis* that display reduced virulence, suggesting that this septin is pivotal for septin‐mediated appressorium penetration in *B. maydis*.

Septins are also regulated post‐translationally. A global analysis of sumoylation, a post‐translational modification that consists of the attachment of the small ubiquitin‐like modifier (SUMO) in *M. oryzae*, for example, suggested that the core septins Sep3, Sep4, Sep5 and Sep6 are sumoylated, and it has been reported that when their consensus sumoylation sites are mutated this leads to reduced virulence and mislocalisation of septins at the base of the appressorium (Liu et al., 2018). However, further direct functional evidence is required to determine the role of sumoylation in septin ring formation.

### 2.2 How do septins mediate penetration of hosts?

The *M. oryzae* septin ring appears to act as a lateral diffusion barrier for the correct positioning of specific actin‐associated proteins, such as Las17 which polymerises F‐actin via the Arp2/3 complex (see Figure 2), gelsolin an actin‐severing protein necessary for correct assembly and disassembly of F‐actin, coronin which promotes normal F‐actin remodelling, and Rvs167 associated with endocytosis in *M. oryzae* (Dagdas et al., 2012; Dulal et al., 2021). Septins recruit, scaffold and organise the F‐actin toroidal network at the appressorium pore, building phosphoinositide linkages with the plasma membrane, supporting linkages between F‐actin and the plasma membrane via Tea1, an ezrin‐radixin‐moesin (ERM) and Kelch protein (Dagdas et al., 2012). Tea1 furthermore demonstrates crosstalk with the cyclic AMP (cAMP)—protein kinase A (PKA) and Pmk1 MAPK pathways and is involved in cell polarisation during appressorium formation (Qu et al., 2022) (see Figure 2).

Moreover, 4D fluorescence imaging has revealed that the F‐actin ring often localises to the periphery of the septin disc prior to septin remodelling and later F‐actin is recruited to the upper surface of the septin ring (Dulal et al., 2020) (see Figure 2). In coronin mutants, F‐actin only loosely associates with the septin ring and is frequently disorganised; additionally, Sep5‐GFP structures have a more heterogeneous morphology and central pores in the rings are not well‐formed (Dulal et al., 2021). Loss of the endocytic protein Pal1, for example, causes an abnormal granular distribution of septins Sep5 and Sep6 at the base of the appressorium and they do not form the septin ring. Pal1 has functions upstream of both the cAMP and Pmk1 MAPK signalling pathways (Chen et al., 2022). In the anthracnose fungus *Colletotrichum gloeosporioides*, a deletion mutant of the F‐actin cross‐linker, fimbrin, affects polarity of the actin cytoskeleton at the hyphal tip and also disrupts the F‐actin ring at the base of the appressorium, preventing appressorium development and thereby affecting pathogenicity (Zhang et al., 2022). In hyphae of *M. oryzae*, fimbrin patches mainly form a subapical endocytic collar, while in appressoria they organise at the periphery of the cell, “sitting” on the F‐actin ring (Li et al., 2020). Without fimbrin, the actin cytoskeleton at hyphal tips fails to assemble the exocyst in a septin‐dependent manner to achieve polarised growth (Li et al., 2020). Taken together, the reports indicate that endocytic proteins are not only necessary for penetration but also for cell‐to‐cell movement and colonisation of the hosts.

The septin lateral diffusion barrier secures correct localisation of the exocyst octameric complex to the appressorium pore, which is necessary for polarised exocytosis in *M. oryzae* and emergence of the penetration peg (Gupta et al., 2015) (see Figure 2). This may be fundamental to pathogenesis, because it has recently been shown that some effector proteins, implicated in suppression of plant immunity, are expressed prior to plant infection and likely secreted at the base of the appressorium at the onset of penetration peg development (Yan et al., 2023). Along with the Spitzenkörper, polarisome, polarity factors and the actin cytoskeleton, the exocyst also plays a key role in polarised growth in filamentous fungi (Araujo‐Palomares et al., 2009, 2011; Delgado‐Álvarez et al., 2010; Garduño‐Rosales et al., 2022; Riquelme et al., 1998, 2014). Exocyst assembly is septin‐dependent and requires regulated synthesis of reactive oxygen species (ROS) in *M. oryzae* (Gupta et al., 2015). Co‐immunoprecipitation of the exocyst proteins Sec6 and Exo84, showed interactions with all four core septin GTPases, as well as Rho1 and Rac1 (Rho GTPases), fimbrin and Pmk1 (Gupta et al., 2015). It has been recently discovered that another ring structure, composed of CK2 proteins, perpendicular to the septin ring also needs to be properly located and assembled in the appressorium to facilitate full penetration peg function and subsequent plant infection in *M. oryzae* (Zhang et al., 2021) (see Figure 2). It has been reported that assembly of the CK2 ring depends on initial assembly of the Sln1‐septin‐exocyst complex and is potentially involved in re‐polarisation of the appressorium (Zhang et al., 2021).

Mutations of two cell cycle GAP‐encoding genes *BUB2* and *BFA1* in *C. orbiculare*, negatively impact the dynamics of septins and the assembly of F‐actin (Fukada & Kubo, 2015). The septin ring in *M. oryzae* assembles after Sln1‐dependent turgor sensing (Ryder et al., 2019) and progression of the appressorium nucleus through S‐phase in *M. oryzae* (Osés‐Ruiz et al., 2017). Sln1 acts via the Pkc1‐dependent cell‐integrity pathway to activate the Nox2‐NoxR NADPH oxidase complex which is necessary for septin ring formation (Ryder et al., 2013) and potentially regulated by the transcription factor BZIP3 (Liu et al., 2022). The transcription factor Tpc1, meanwhile, regulates *NOXD* expression, the p22^phox^ subunit of the NADPH oxidase complex by interacting with Mst12, a regulator of the Pmk1 pathway (Galhano et al., 2017). In addition, septin ring assembly requires Cdc42, a small Rho GTPase that acts as a polarity determinant, and Chm1 (an ortholog of Cla4), a p21‐activated kinase that phosphorylates septins (Dagdas et al., 2012) (see Figure 2).

In *M. oryzae*, ROS generation is required for both F‐actin and septin ring assembly and may be necessary for actin‐mediated remodeling of disk‐like septin structures into rings. Gelsolin, for example, may be directly controlled by redox regulation (Ryder et al., 2013). The Nox2/NoxR complex is essential for septin‐mediated F‐actin assembly, which translates into initiation of polarised growth of the penetration peg, and Nox1 is required for maintenance of penetration peg elongation (Ryder et al., 2013). Furthermore, Nox2 interacts with Rac1, which is involved in polarised growth and F‐actin dynamics, with gelsolin being a downstream effector (Arcaro, 1998; Chen et al., 2008). The Sep4 septin of *Botrytis cinerea* forms part of the diffusion barrier necessary for melanin and chitin accumulation in hyphal tips, appressorium formation and host penetration of the grey mould fungus (Feng et al., 2017). Inhibition of ROS production in *B. cinerea*, causes disordered assembly of Sep4 and failure to form infection cushions, meaning that proper Sep4 assembly, regulated by controlled ROS production, is essential for initiation of infection structure formation and infection (Feng et al., 2017; Hou et al., 2020). Jar1 controls ROS production by orchestrating global expression of necessary genes, and deletion mutants of *JAR1* show defects in conidiation, appressorium formation, stress adaptation, infection cushion formation and virulence, but the mutation promotes sclerotium production (Hou et al., 2020).

*Verticillium dahliae* is a root‐infecting pathogen that infects cotton by developing a penetration peg from its hyphopodium infection structure. The septin ring separates the hyphopodium from the invasive hypha. NADPH oxidases regulate septin ring organisation. A study revealed that several signal‐peptide containing secreted proteins showed ring signal accumulation at the penetration interface surrounding the hyphal neck. This localisation pattern was not present in Δ*sep5*, Δ*sec22*, Δ*syn8* and Δ*exo70* mutants, suggesting that septins and the secretory pathway are important for delivery of these secreted proteins to the penetration interface (Zhou et al., 2017).

Some multiple auxiliary activity family 9 (*AA9*) genes are known to be required for the virulence of plant pathogenic fungi. Aa91, for example, is a secretory protein which localises to the apoplast and is essential for pathogenicity of *V. dahliae*. Interestingly, the Δ*aa91* mutant cannot produce a penetration peg or assemble a septin ring at the base of the hyphopodium (Chen et al., 2022). Moreover, the secretory protein Scp41 localises to the base of the hyphopodium and shows a ring signal surrounding the hyphal neck. Scp41 functions as an intracellular effector that promotes *V. dahliae* virulence by targeting and inhibiting the transcription factor activity of plant immune regulators BP60g, SARD1 and GhCBP60b (Qin et al., 2018).

### 2.3 How do septins focus fungal invasive growth in host tissue?

Cell‐to‐cell movement by *M. oryzae* requires a second specific morphogenetic switch, whereby the hyphal tip swells isotropically when it encounters a pit field and then drastically constricts to around 360 nm (the diameter of a pit field) before re‐polarising to develop a new invasive hypha that enters the neighbouring cell (Kankanala et al., 2007). Live‐cell imaging of this process has shown that invasive hyphae reach ~5.0 μm in diameter once they become swollen and constrict to 0.3–0.8 μm in diameter when they move through pit fields (Sakulkoo et al., 2018). Due to the similarity in the developmental process that takes place when an appressorium develops a penetration peg, the infection structure involved in cell‐to‐cell movement has been called a “transpressorium”, a term first coined by Liese and Schmid (1964). Both appressorium and transpressorium development are likely to be morphogenetically related processes (Cruz‐Mireles et al., 2021). In *M. oryzae* transpressorium development also appears to be a septin‐mediated mechanism that requires actin cytoskeleton remodelling (Sakulkoo et al., 2018). Septin mutants are, for example, impaired not only in appressorium function, but also show defects in cell‐to‐cell movement in the rare instances that invasive hyphae are formed. To address this question more directly, conditional mutants of *M. oryzae* septins will need to be generated, which is now possible using CRISPR‐Cas9 mutagenesis (Foster et al., 2018).

### 2.4 Do septins influence the morphology of hyphae and infectious propagules by pathogens?

The necrotrophic plant pathogen *Fusarium graminearum* is the causal agent of fusarium head blight (FHB) and contains seven putative septin‐encoding genes: Cdc3, Cdc10, Cdc11, Cdc12, AspE, AspE2 and Hyp7A (see Table 1) (Pan et al., 2007). The functional characterisation of the four core *F. graminearum* septins, Cdc3, Cdc10, Cdc11 and Cdc12, revealed that Δ*cdc3*, Δ*cdc11* and Δ*cdc12* mutants showed reduced growth, conidiation and virulence. By contrast, Δ*cdc10* mutants had no significant effect on pathogenicity. Additionally, all septin mutants showed morphological asexual and sexual spore defects and an abnormal distribution of nuclei, suggesting an involvement in nuclear division. Microscopy revealed their function in septum formation (Chen et al., 2016). Interestingly, the four core septins interact with the end‐binding protein 1 (EB1), which is known to regulate microtubule dynamics, cell polarisation and chromosome stability in yeast. EB1 deletion mutants exhibited twisted hyphae, hyperbranching and curved conidia, supporting the proposed function of Eb1 in regulating cell polarity. The organisation of microtubules, myosin and actin was altered as well (Liu et al., 2017). Studies within the filamentous fungus *F. asiaticum* confirmed the importance of Cdc3 and Cdc12 for hyphal and colony morphology, growth, conidiation, septation in hyphae and conidia and nuclear distribution, as well as for virulence. Additionally, it was shown that in Δ*cdc3‐* and Δ*cdc12* mutant‐infected wheat spikelets, levels of the mycotoxin deoxynivalenol decreased. Sexual reproduction in wheat kernels was also reduced. In both deletion mutants, conidia and hyphae formed internal large lipid droplets, indicating that the lipid metabolism was additionally affected (Zhang et al., 2017).

*B. maydis* also requires core septins, Cdc3, Cdc10, Cdc11 and Cdc12 (see Table 1), to develop reproductive propagules (Tsuji et al., 2022; Zhang et al., 2020). Core septin mutants produce conidia and ascospores with aberrant morphologies, while a mutant of the non‐core septin Cdc100 has a wild‐type phenotype (Zhang et al., 2020). Colony growth is most affected in the double mutants Δ*cdc3*Δ*cdc10* and Δ*cdc3*Δ*cdc11*, and although all core septins are important for reproductive propagule formation, only Δ*cdc10* and Δ*cdc3*Δ*cdc10* double mutants showed decreased virulence (Zhang et al., 2020).

*Cryphonectria parasitica*, the causal agent of chestnut blight, requires the septin Sep1 (an ortholog of the *M. oryzae* Sep5) for pathogenicity, as it regulates production of normal rod‐shaped conidia, and its loss causes growth delay, hyperbranching, a decrease in aerial mycelium production, hyperpigmentation and hypersensitivity to ROS (Jo et al., 2019).

In *V. dahliae*, the four core septins, Sep3, Sep4, Sep5 and Sep6 (see Table 1), play different roles in regulating microsclerotial development, melanin synthesis and stress responses, and all four mutants show abnormal chitin distribution and defects in virulence (Wang et al., 2022). Deletion mutants of kinesin 2 (Kin2) also showed defects in virulence including mis‐localisation of the septin ring. Kin2 is associated with vacuole formation, which is known to play a critical role in normal development of infection structures. Δ*kin2* mutants lacked a large basal vacuole and failed to generate concentrated lipid droplets. Additionally, Kin2 was important for conidiation, mycelium growth and branching of hyphae in *V. dahliae* (Yang et al., 2022). Taken together these studies suggest that septins play important roles in infectious propagule development by pathogenic ascomycete fungi. However, it is unclear which hetero‐oligomeric septin structures are assembled in these species, given the discrepancies observed in mutant phenotypes of individual septins. A more systematic analysis of septin function is required along with an analysis of the structural dynamics and interactions of septins in a more diverse set of pathogens to make more general conclusions.

## 3 SEPTINS IN THE BASIDIOMYCETE *USTILAGO MAYDIS* AND THEIR ROLE IN PLANT PATHOGENESIS

The corn smut fungus *Ustilago maydis* is the only basidiomycete in which the role of septins during pathogenesis has been studied to date. This maize pathogen infects plants following fusion of compatible haploid budding yeast cells, called sporidia, to form dikaryotic filaments which then develop appressoria to penetrate the intact plant surface and colonise plant tissue (Boyce et al., 2005). *U. maydis* possesses four septins designated Sep1, Sep2, Sep3 and Sep4 (see Table 1). These septins assemble into bud neck collars, band‐like structures at the polarised growing hyphal tip as well as forming long septin filaments near the cell cortex, stretching from pole to pole. Strikingly, septins can also spontaneously self‐assemble in the cytoplasm of *U. maydis*, where they can either form actin‐associated linear filaments in growing cells, or rings during stationary phases. When cells are treated with latrunculin A, an actin depolymerising drug, linear filaments disassemble, whilst in growing cells septin ring formation is affected (Aschenbroich et al., 2019). This phenomenon has also been observed in mammalian fibroblasts (Kinoshita et al., 2002).

Single deletion mutants showed how important septins are for polarised growth and morphogenesis in general. Without septins, *U. maydis* cells lose their polarity and thus their elongated shape, so the central region of the cell becomes wider. The application of drugs that affect synthesis of the cell wall and exocytosis, revealed that septin mutants are more sensitive towards those drugs than the wild‐type, an effect which could be attenuated by an osmotic stabiliser, suggesting their importance in proper formation of the cell wall.

Interestingly, unlike other plant pathogens, septin mutants in *U. maydis* retain virulence and play only a minor role during maize infection (Alvarez‐Tabares & Perez‐Martin, 2010). A Δ*sep3* mutant, for example, produces slightly fewer tumours inside plants but filamentous cells also display some morphological defects *in planta*. Furthermore, septins negatively affect the differentiation of hyphae into teliospores and their subsequent germination (Boyce et al., 2005). In addition, it is known that the MAPK signalling cascade and the cAMP‐PKA‐dependent pathway impact morphological changes (D'Souza & Heitman, 2001). Both pathways regulate dimorphism in *U. maydis* and convert environmental signals, from lipids or pheromones, released from cells of the opposite mating types to induce transition from the haploid yeast‐like budding growth to filamentous dikaryotic growth (Boyce et al., 2005). Transcript levels of *SEP3* in Δ*ubc1* mutants, lacking the regulatory subunit of cAMP‐dependent PKA, were downregulated and revealed a link between the cAMP signalling pathway, septins, and morphological changes in *U. maydis*. Both Δ*ubc1* mutants and Δ*sep3* mutants show a multiple budding phenotype due to the defect in the separation of mother and daughter cells (Boyce et al., 2005; Larraya et al., 2005).

In *S. cerevisiae*, Cdc42 marks the incipient bud site and organises the assembly of a septin ring which then defines the future mother bud neck (Longtine & Bi, 2003). Deletion mutants of Cdc42 in *U. maydis* similarly show a cell separation defect during budding, however, bud formation and morphology are not altered. Interestingly, deletion of both Rac1 and Cla4 affects bud formation and might be necessary for organisation of the septins required for bud initiation (Mahlert et al., 2006). Another protein important for the site of polarised growth is Tea1; Δ*tea1* mutants bud from both cell poles. Tea1 interacts with itself and localises to sites of polarised growth, where it may act as a scaffold for assembly of proteins such as septins that determine the site of polarised growth (Woraratanadharm et al., 2018), whereas the protein Mes1 is essential for strong polarised hyphal growth but not for yeast‐like growth. In Δ*mes1* mutants the septin ring is absent from the bud tip but not the bud neck, suggesting that cell division is not affected whereas polarised growth is impacted (Cánovas, 2022).

Generally, *U. maydis* displays two distinct classes of septa. The primary septum of haploid budding cells and septa formed during growth *in planta* are coupled to mitosis and do not require the Cdc42 signalling module. The secondary septum of haploid budding cells and septa of infectious hyphae, are also independent of mitosis but require the Cdc42 signalling module (Freitag et al., 2011). While the phosphorylation of septins via Cla4 proved to be important for primary septum formation, the Ste20‐like kinase Don3, phosphorylates septins and triggers formation of the secondary septum (Leveleki et al., 2004). First, the essential light chain of type II myosin Cdc4 associates with the septin collar at the secondary septum site. Don3 then triggers septin reorganisation and incorporation of Cdc4 into the actomyosin ring, which also contains the F‐BAR domain protein Cdc15 (Aschenbroich et al., 2019; Böhmer et al., 2009). From studies in the fission yeast *Schizosaccharomyces pombe* it is known that Cdc15 connects the actomyosin ring to the plasma membrane to co‐ordinate invagination of the plasma membrane (Aspenström et al., 2006; Chitu & Stanley, 2007). In *U. maydis*, reassembly of septin filaments into a ring‐like structure fails in the absence of either F‐actin or Cdc15. This suggests that septin ring formation itself depends on a functional contractile actomyosin ring (Aschenbroich et al., 2019). Cdc42 is also crucial for the assembly of the actomyosin ring, but Don3 activity precedes those of Cdc42 (Böhmer et al., 2008). The impact of Don3 triggers a dynamic rearrangement of higher‐order septin structures. Don3 is also required for assembly of a functional fragmentation zone, which is the space between the primary and secondary septum. In this context, Don3 influences the cell cycle‐dependent unconventional secretion of chitinase Cts1 via the fragmentation zone (Böhmer et al., 2009).

To penetrate the plant cuticle, infectious hyphae of *U. maydis* eventually differentiate into appressoria. The growth of the infectious hyphae requires regular formation of retraction septa leaving empty sections behind. Environmental signals then trigger formation of an appressorium at the tip cell. The diaphanous‐related formin Drf1, acts as an effector of a Cdc42 GTPase signalling module, which also consists of the Cdc42‐specific guanine nucleotide exchange factor Don1 and Don3. The deletion of *DRF1*, *DON1* or *DON3* abolishes formation of retraction septa. Appressorium formation in these mutants is not completely blocked, but infection structures are only found at the tip of short filaments. This suggests that retraction septa are necessary for appressorium formation in elongated infectious hyphae. Also, septin ring assembly and actomyosin ring formation depend on Drf1, and Δ*drf1* mutants penetrate the plant tissue less frequently. The cell‐cycle arrest is subsequently released after plant penetration. Hyphal septation during the biotrophic phase is thereafter independent of the Don1/Cdc42/Drf1/Don3 signalling module (Freitag et al., 2011).

In addition to questions regarding subcellular localisation of septins at distinct sites, it has also been illuminating to determine how intracellular transport of septins operates and how they then initially assemble. Endosomes can transport lipids, proteins and mRNA over long distances by shuttling along microtubules. Live‐cell imaging of polarised hyphae shows that septins are transported via endosomes along microtubules and that this is crucial for formation of higher‐order structures in *U. maydis*. Additionally, septin endosomal localisation depends on the mRNA‐transport protein Rrm4 and on Upa2, with the latter functioning as a core component of endosomal mRNA transport, providing scaffolding function and associating mRNAs, Pab1 and Rrm4 on endosomes. Strikingly, the endosomal transport of septins depends on each of the four septins, suggesting that heteromeric complexes are already assembled at the membranous surface of endosomes. Furthermore, endosomal trafficking of all four septin mRNAs is required for endosomal localisation of their translation products. This indicates that local translation may promote assembly of newly synthesised septins into heteromeric structures at the surface of endosomes, supporting the long‐distance transport of septins to the growth poles, and thus, the efficient formation of the septin cytoskeleton (Baumann et al., 2014; Jankowski et al., 2019; Zander et al., 2016).

## 4 SEPTINS AS A TARGET FOR ANTIFUNGAL PLANT PROTECTION APPROACHES

Given the essential role of septins in appressorium‐mediated plant pathogenic infection, blocking their assembly into higher order structures is a potential target for fungicides to prevent host penetration and disease—not least because plants do not possess septins. A recent study showed that biosynthesis inhibitors of very‐long‐chain fatty acids (VLCFA) with more than 20 carbons, prevent the recruitment of septins to curved plasma membranes in *M. oryzae*. Therefore, the septin ring cannot assemble and host penetration is prevented (He et al., 2020). Interestingly, the herbicides metazachlor, cafenstrole and diallate, successfully inhibit production of VLCFAs in the fungal pathogen and were shown not only to prevent rice blast disease, but also prevent infection by a wide range of other fungal pathogens, such as *B. maydis* (southern‐corn leaf blight disease), *Blumeria graminis* (wheat powdery mildew disease) and *Metarhizium acridum* (an entomopathogenic fungus), without affecting the hosts. The findings of He et al. (2020) demonstrate the existence of a mechanism underlying septin assembly at plasma membranes during infection structure formation in fungi, that may provide a means of controlling diverse diseases of plants and animals caused by fungi.

Interestingly, melatonin can inhibit 13 plant pathogens, including fungi such as *M. oryzae* as well as oomycetes. Once melatonin enters the pathogen it has been reported to bind to the Mps1 MAPK which regulates cell integrity inhibiting Mps1 phosphorylation and suppressing infection in both rice and barley seedlings (Li et al., 2022; Sakulkoo et al., 2018; Xu et al., 1998). Melatonin delays conidial germination, resulting in swollen germ tubes and malformed appressoria, and when applied directly to appressoria it has been shown to inhibit septin ring formation in *M. oryzae* (Li et al., 2022). Melatonin could therefore be a low‐cost alternative to combat fungal plant pathogens and the development of an eco‐friendly melatonin derivative—that does not have any effects on human physiology—through systematic modifications has been proposed (Li et al., 2022).

Finally, it is known that chitosan causes massive membrane permeabilisation that inhibits growth of fungi, which could make it a powerful antifungal agent. It was recently found that chitosan prevents septin‐mediated re‐polarisation of the appressorium by affecting NADPH oxidase‐dependent synthesis of ROS, making it an attractive agent to prevent rice blast disease because it acts prior to leaf penetration (Lopez‐Moya et al., 2021). To be effective, chitosan requires the Pkc1‐dependent cell wall integrity pathway, the Mps1 mitogen‐activated protein kinase and Nox1 (Lopez‐Moya et al., 2021). In addition to being a promising agent for development of a broad‐spectrum, fungicide, chitosan effectively improves physiological properties of plants and is a sustainable and biodegradable product (Malerba & Cerana, 2016; Sharif et al., 2018).

## 5 FURTHER RESEARCH PERSPECTIVES

After their initial discovery in *S. cerevisiae*, septins have been identified in many eukaryotic organisms, where they fulfil diverse functions in cell organisation and morphogenesis (Li et al., 2021; Ngo & Mostowy, 2019; Shuman & Momany, 2021). In plant pathogenic fungi septins are strongly associated with infection‐associated development and therefore crucial to the ability of fungi to invade living host tissues. Septins are critical for cell shape changes and, therefore, important for initiation of polarised growth. Septins function as scaffolds to recruit proteins, but they also form diffusion barriers to create subcellular compartments and discrete membrane domains. However, it remains unknown how septins are precisely recruited to sites of polarised growth, especially at the host–pathogen interface, where they fulfil such important roles. Does the recruitment of septins exclusively depend on membrane curvature, for instance, or does it depend on other proteins, such as BAR domain proteins which can generate and sense membrane curvature that might be pre‐requisite for septin aggregation? Once septin assemblies are made do they fulfil a wider function than just the regulation of polarised growth? Is it possible that septins actually act as key organising centres for cell signalling, acting for example, to spatially and temporally regulate the deployment of virulence factors, such as effector proteins that suppress plant immunity and enable proliferation of fungal pathogens in host cells? El‐Mansi et al. (2023) have, for example, recently demonstrated that septins can be key organising centres of a wider range of cellular processes than originally envisaged.

The structure of septin hetero‐oligomers has been thoroughly analysed in model species (Bertin et al., 2008; DeMay et al., 2011; McMurray & Thorner, 2009; Mendonça et al., 2021; Weems & McMurray, 2017; Woods & Gladfelter, 2021), but nonetheless, how septins are assembled and distributed has not been deciphered in any fungal pathogen to date. It remains unclear, for instance, how septins assemble during ring formation, as well as how they assemble in filaments at the cell cortex during hyphal growth. It is known that septin heteromeric complexes can assemble at the surface of endosomes, but their exact composition needs to be assessed. Similarly, it is not known how septin hetero‐oligomers are dynamically assembled and depolymerised during septin reorganisation. Conditional mutants would provide valuable insight into septin assembly/disassembly events during infection, which has proved impossible to study with deletion mutants. Gaining this kind of knowledge could be critical in determining how to inhibit septin assembly at specific points during infection, such as primary host infection or during invasive growth, thus preventing disease development. Live‐cell imaging and cryo‐electron tomography techniques are powerful tools to address the dynamics of septin assembly. Based on the crystal structure of septin hetero‐oligomeric complexes, new chemical compounds could, for example, be developed to fight plant diseases. The absence of septins in plants provides a unique opportunity, since off‐target effects will not be present in the host. Assembly of septins also requires post‐translational modifications such as phosphorylation and dephosphorylation, however, and determining which kinases and phosphatases regulate septin higher‐order structures also remains a future challenge.

Finally, the class V septins, or non‐core septins, are exclusive to some filamentous fungi and protists (Shuman & Momany, 2021), but their roles remain unclear at this time. If they are subsequently shown to also be necessary for pathogenesis, then they would be valuable targets for anti‐fungal strategies due their specificity. However, it is not clear how they interact with other septins, in distinct heteropolymers, or whether they form completely different homopolymeric assemblages. Determining the function of these septins, such as Sep7 and Sep8 in *M. oryzae*, is a critical challenge for determining the role of septins in fungal pathogenesis.

In summary, septins play highly diverse roles in fungal pathogens, affecting appressorium and hyphopodium development, conidiation, hyphal growth, transpressorium function and invasive growth. There is, however, an increasing body of evidence that the functions of septins in the regulation of infection‐related development are likely to be widespread among the most important crop pathogenic species and therefore worthy of more intense and systematic study in future. If detailed comparative analysis of septin function can be carried out, alongside selection of specific inhibitors of septin assembly, then this could lead to entirely novel anti‐fungal strategies which could be widely deployed.

## FUNDING INFORMATION

This research was funded by The Gatsby Charitable Foundation, The Biotechnology and Biological Sciences Research Council Institute Strategic Project in Plant Health (BBS/E/J/000PR9797) and The European Research Council Advanced Grant SEPBLAST awarded under the UKRI guarantee scheme (EP/X022439/1).

## CONFLICT OF INTEREST STATEMENT

The authors declare no competing interests.

## Data Availability

Data sharing not applicable to this article as no datasets were generated or analysed during the current study.
